# Development and Optimization of Naringenin-Loaded Chitosan-Coated Nanoemulsion for Topical Therapy in Wound Healing

**DOI:** 10.3390/pharmaceutics12090893

**Published:** 2020-09-20

**Authors:** Sabah H. Akrawi, Bapi Gorain, Anroop B. Nair, Hira Choudhury, Manisha Pandey, Jigar N. Shah, Katharigatta N. Venugopala

**Affiliations:** 1 Department of Pharmaceutical Sciences, College of Clinical Pharmacy, King Faisal University, Al-Ahsa 31982, Saudi Arabia; kvenugopala@kfu.edu.sa; 2 School of Pharmacy, Faculty of Health and Medical Sciences, Taylor’s University, Subang Jaya, Selangor 47500, Malaysia; bapi.gorain@taylors.edu.my; 3 Centre for Drug Delivery and Molecular Pharmacology, Faculty of Health and Medical Sciences, Taylor’s University, Subang Jaya, Selangor 47500, Malaysia; 4 School of Pharmacy, International Medical University, Bukit Jalil, Kuala Lumpur 57000, Malaysia; hirachoudhury@imu.edu.my (H.C.); manishapandey@imu.edu.my (M.P.); 5 Department of Pharmaceutics, Institute of Pharmacy, Nirma University, Ahmedabad 382481, Gujarat, India; jigar.shah@nirmauni.ac.in; 6 Department of Biotechnology and Food Technology, Durban University of Technology, Durban 4001, South Africa

**Keywords:** chitosan-coating, nanoemulsion, Box–Behnken model, cytotoxicity study, abrasion wound model, naringenin

## Abstract

The potential role of naringenin (NAR), a natural flavonoid, in the treatment of chronic wound has prompted the present research to deliver the drug in nanoemulsion (NE) form, where synergistic role of chitosan was achieved through development of chitosan-coated NAR NE (CNNE). The NE consisted of Capryol 90, Tween 20 and Transcutol P, which was fabricated by low-energy emulsification method to encapsulate NAR within the oil core. The optimization of the formulated NEs was performed using Box–Behnken statistical design to obtain crucial variable parameters that influence globule size, size distribution and surface charge. Finally, the optimized formulation was coated with different concentrations of chitosan and subsequently characterized in vitro. The size of the CNNE was found to be increased when the drug-loaded formulation was coated with chitosan. Controlled release characteristics depicted 67–81% release of NAR from the CNNE, compared to 89% from the NE formulation. Cytotoxicity study of the formulation was performed in vitro using fibroblast cell line (NIH-3T3), where no inhibition in proliferation of the cells was observed with CNNE. Finally, the wound healing potential of the CNNE was evaluated in an abrasion-created wound model in experimental animals where the animals were treated and compared histologically at 0 and 14 days. Significant improvement in construction of the abrasion wound was observed when the animals were treated with formulated CNNE, whereas stimulation of skin regeneration was depicted in the histological examination. Therefore, it could be summarized that the chitosan coating of the developed NAR NE is a potential platform to accelerate healing of wounds.

## 1. Introduction

Development of chronic wounds is a consequence of failure in the normal wound healing process in an orderly and timely manner [1]. The self-regenerative capacity of cutaneous wounds is lost in chronic wound conditions, which may not be able to achieve anatomical and functional integrity of the damaged tissue by the succession complex stages, such as hemostasis, inflammation, proliferation and resolution within the period of six weeks of medical therapy [2,3]. Therefore, the chronic wounds remained in the inflammatory phase in venous leg ulcers, diabetic foot ulcers and pressure ulcers [4]. The incidences of chronic wound cases are increasing sharply every year, predominantly in the elderly [5]. Improper control of chronic wound patients has significant influence on the health, leading to amputation of the extremities, which impact on the quality of life of the patients and sometime leading to death [1]. Physiological management of chronic wounds could effectively be managed by topical therapies, where control of acidic pH, proper level of moisture, formation of biofilms by the pathogenic bacterial growth and temperature need to be maintained using a biocompatible environment [5]. However, numerous researches are ongoing in different parts of the world to develop successful wound dressings towards acceleration of the healing process [6,7,8]. It is important to mention that a suitable wound healing material should possess certain characteristics, viz. biocompatibility and permeability of gases for keeping the wound moistened with a barricade to invading microorganisms [9]. Additional support for developing the regeneration capacity of the wound environment by the wound healing material could help in proliferation of cells for speedy resolution in the healing process [10].

Recent researches towards development of wound healing dressings are progressing by the use of natural components, as nature is considered the richest source of phytochemicals for the treatment of numerous ailments [11]. These phytoconstituents are known to possess antioxidant, antimicrobial, anti-inflammatory and angiogenic properties with cell signaling factors which promote the healing process [11]. A widely available polyphenol in citrus fruits, naringenin (NAR), is known to possess antioxidant, antimicrobial and anti-inflammatory characteristics [12,13]. Chemically it is 4,5,7-trihydroxy-flavanone and belongs to the flavonoids category formed by the hydrolysis of narirutin. Naringenin-loaded nanoparticles have also been widely investigated for various applications [14,15,16]. Additionally, an amino sugar composed polysaccharide biomaterial, chitosan, has also been widely used in different pharmaceutical dosage forms due its biodegradable and biocompatible characteristics [17]. This natural linear polymer comprised of glucosamine and *N*-acetyl-d-glucosamine linked by β (1→4) glycosidic bonds and is obtained by partial deacetylation of chitin [18]. Presence of hydroxyl and amine groups on the surface of chitosan undergo protonation at low pH, thereby positive charge of the molecule helps to create electrostatic interaction with the negatively charged components of the cells and mucous, thereby possessing strong mucoadhesive force [19]. Additionally, formation of hydrophobic interaction and hydrogen bonds facilitates the mucoadhesive property of chitosan [20]. Thus, this low toxic and immune responsive material had been suggested to create an ideal cellular microenvironment to regenerate body injuries through secure adherence at the affected area [21].

Therefore, the present research is attempting to develop an ideal wound dressing with NAR and chitosan for the effective healing of wounds. In order to achieve this goal, we have selected the nanoemulsion (NE) dosage form to deliver the hydrophobic component NAR within the oil core of oil-in-water type NE, where chitosan was used to coat the formed oil globules. Delivering hydrophobic components using this NE platform makes this nanoformulation a widely explored tool, where the dispersed droplets of 10–200 nm promotes penetration of the drugs for therapeutic responses [22,23]. Incorporation of chitosan in the formulation is aimed towards overcoming its limitations in topical application, which will allow the formed gel to persist at the site of application for longer durations [24]. Thus, the NE formulation was developed using Capryol 90, Tween 20 and Transcutol P using a low-energy emulsification method. Finally, the optimized batch of the NE was determined using the Box–Behnken statistical design. Upon incorporation of the drug within the NE, the chitosan solution was used to develop chitosan-coated NAR NE (CNNE). The developed formulations were characterized and analyzed for in vitro release pattern of NAR from the CNNE. Finally, cytotoxicity of the formulation was evaluated in vitro in the fibroblast cell line and finally wound-healing characteristics were evaluated *in vivo* in abrasion-created wound models on experimental animals to establish superiority of the formulated CNNE in wound healing.

## 2. Materials and Methods

### 2.1. Materials

NAR (purity > 95%), Solutol HS15, Tween 20, Tween 80, Chremophor EL, PEG 400, propylene glycol, 3-(4,5-dimethylthiazol-2-yl)-2,5-diphenyltetrazolium bromide (MTT) and chitosan (low-molecular weight) were obtained from Sigma Aldrich (St. Louis, MO, USA). Plurol Oleique, Labrafac, Transcutol P, Labrafil M1944 CS, Labrafil M2125 CS, Labrasol, Lauroglycol 90 and Capryol 90 were gifted by Gattefosse, France and Sefsol 218 was gifted by Nikko Chemicals Co. Ltd., Tokyo, Japan. Invitrogen (Carlsbad, CA, USA) supplied Roswell Park Memorial Institute (RPMI)-1640, fetal bovine serum (FBS)-heat-inactivated and antibiotics including other materials for cell culture. Sunflower oil, oleic acid, castor oil and peanut oil were obtained from local suppliers. Deionized water and analytical grade chemicals were used in each step of the experiments, where the chemicals were used without further purification.

### 2.2. Screening of Nanoemulsion Components

During selection of components for the development of NAR NE, different oils, surfactants and co-surfactants were used to evaluate NAR solubility. In due course of solubility determination, an excess quantity of NAR was added in 2 mL of each component followed by vortex mixing for 5 min. Later, the tubes were shaken at 100 rpm in an isothermal shaker bath for 72 h to reach equilibrium solubility [25]. The contents were then centrifuged at 5000 rpm for 30 min and the precipitates were solubilized using methanol and analyzed using a UV-visible spectrophotometric method at lambda max of NAR, 289 nm. The standard curve for the analysis of NAR concentration is represented in Supplementary Figure S1.

### 2.3. Selection of Surfactant and Co-Surfactant Ratio Using Pseudoternary Phase Diagrams

An aqueous titration method was adopted to determine the NE region in the constructed pseudoternary diagrams using different ratios of selected surfactant and co-surfactant. Thus, the pseudoternary diagrams represent the NE regions using Capryol 90, mixture of surfactant and cosurfactant (Smix) at different volume ratios (3:1, 2:1, 1:1, 1:2, 1:3) and water. The ratios were selected with decreasing concentration of surfactant with respect to co-surfactant or vice versa in order to cover the entire range. The selected ratio of Smix were specifically combined with different ratios of oil (1:9, 2:8, 3:7, 4:6, 5:5, 6:4, 7:3, 8:2 and 9:1) to determine the NE region. The selected combinations of oil and Smix secure maximum ratios for the NE zone determination [26]. In this aqueous titration method, the aqueous phase was slowly added to the mixture of oil and Smix dropwise with vigorous stirring. Formation of clear, transparent to translucent or slightly bluish appearance of the formulation was considered as NE [27]. Finally, the pseudoternary phase diagrams were plotted using oil phase, Smix and aqueous phase at the three-axis, where the circular dots were representing the NE region.

### 2.4. Preparation of Blank Nanoemulsion

For the low-energy emulsification technique, the aqueous titration method was used to develop the blank NEs using selected components and ratio following solubility study and pseudoternary phase diagram. Capryol 90 and selected ratio of Smix were mixed together followed by dropwise addition of aqueous phase with continuous mixing using vortex mixer to form a clear, transparent and homogeneous NE. Further, the developed NEs were analyzed using Zetasizer (Nano-ZS90, Malvern Instruments, Worcestershire, UK) for the droplet size, polydispersity index (PDI) and zeta potential [28]. Thermodynamic stability of the formulated NEs were performed by centrifugation at 5000 rpm for 30 min, and passing through three cycles of heating and cooling (at 40 °C and 4–8 °C) and freeze-thaw cycles (−20 °C and 25 °C) [29].

### 2.5. Optimization of NRG Nanoemulsion Using Box–Behnken Statistical Design

Three-factor three-level Box–Behnken statistical design (Design Expert, version 12; State-Ease Inc., Minneapolis, MN, USA) was used for the optimization of the blank NE formulations. Based on our previous research and literature data, the highest and lowest concentrations were incorporated for Capryol 90, Smix (Tween 20 and Transcutol P) and purified water for identification of the ideal ratio of the components using the Quality by Design (QbD) technique [26]. There were 17 batches suggested of different combinations of oil, Smix and water in their low (−1), medium (0) and high (+1) levels to be developed and analyzed for the three dependent variables such as droplet size, PDI and surface charge (Table 1). Subsequently, the results of dependent variables from the actual batches were incorporated for the optimization of NE formulations. The statistical design generated 100 theoretical runs to achieve the optimized formulation by evaluating the effect of independent variables. Analysis of variance (ANOVA) was used for the statistical analysis. Interaction between the independent variables was evaluated from the generated perturbation plots, contour plots and 3D surface plots [30]. The software generated quadratic Equation (1) is represented below:

Y = b0 + b1 × A + b2 × B + b3 × C + b12 × AB + b13 × AC + b23 × BC + b11 × A2 + b22 × B2 + b33 × C2(1)

where measured response is represented by Y, b0 is the intercept and the regression coefficients are represented by b0 to b3 for the model term of A, B and C, respectively.

### 2.6. Development of Drug-Loaded Nanoemulsion

Finally, NAR-loaded oil-in-water NEs were formulated consisting of similar proportions of oil, Smix and aqueous phases of the optimized formulation. Similar to the previous method, the low energy emulsification technique was used to formulate the drug-loaded NE formulation where 20 mg NAR was dissolved in the oil phase followed by the addition of Smix and aqueous phase to formulate 10 mL of the NE formulation.

### 2.7. Preparation of Chitosan-Coated Naringenin Nanoemulsion

Three different batches of CNNE were formulated with varying percentages of chitosan. Firstly, the chitosan solution was prepared in 1% acetic acid in purified water. Later 0.5%, 0.75% and 1% of chitosan was used to formulate the CNNEs by replacing the aqueous phase of the formulations by the chitosan solutions followed by mixing vigorously for 10 min [31].

### 2.8. Determination of Morphology of the Developed CNNE

Transmission electron microscopic evaluation was performed to determine the morphology of the formulated CNNE. The microscopic image of the CNNE also provides information on the size of the dispersed droplets and the inner structure of the NE carrier. To carry out the analysis, a drop of the diluted sample (100 times) was kept on a 300 mesh carbon-coated copper grid. Finally, 2% phosphotungstic acid was used to negatively stain the droplets, which was examined under the microscope operated at 100 kV [32].

### 2.9. Determination of the pH and Viscosity of the CNNEs

The pH of NE and CNNEs was measured by using a Sartorius PB-10 pH meter at 25 °C. Simultaneously, viscosity of the formulated NEs and CNNEs was measured by using a Brookfield viscometer using spindle 63 at room temperature. All the measurements were made in triplicate.

### 2.10. Ex Vivo Mucoadhesive Strength

**Figure 1:**
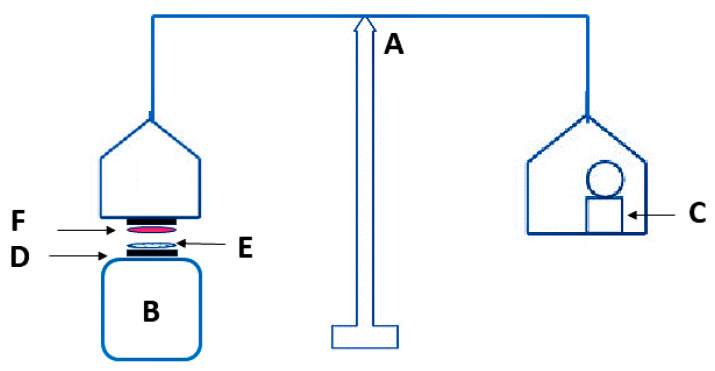
Modified balance assembly for mucoadhesion measurement. **A**: balance; **B**: beaker; **C**: weight; **D**: double sided adhesive tape; **E**: formulation; and **F**: goat skin.

Ex vivo mucoadhesive strength of developed formulations was evaluated in triplicate using the modified balance method as elaborated by Pendekal and Tegginamat [33]. A goat skin was obtained from the local slaughter house, which was cleaned and soaked in phosphate buffer saline (pH 7.4) prior to measurement. A piece of skin was attached to the base of the pan of balance using double-sided adhesive tape. Each formulation was placed on the back side of a beaker as shown in Figure 1. A contact force of 20 g was applied for 90 sec, subsequently the applied force was removed and the weight for detachment of skin from formulation was considered as mucoadhesive strength of formulation. Force of adhesion was calculated by following Equation (2):

(2) $$\mathrm{Force}\;\mathrm{of}\;\mathrm{adhesion}\;\left(N\right)=\frac{Mucoadhasive\;strength\;\left(g\right)}{1000}\times 9.8$$

### 2.11. In Vitro Release Study for NAR

The dialysis bag method was used to estimate the release for NAR in a time dependent manner. An appropriate amount of NEs containing 5 mg of drug was placed in a dialysis bag (12 kDa cut off) and closed properly from both ends using dialysis tubing closer [34]. Phosphate buffer saline (pH 7.4) was used as dissolution media and the dialysis bag was submerged in 100 mL of media with 100 rpm stirring at 37 °C temperature. Subsequently, 1 mL samples were withdrawn at definite time-points and the same amount of fresh media was added to maintain sink condition. Percentage release of samples was estimated at 289 nm using UV spectrophotometer. The experiment was performed in triplicate.

### 2.12. In Vitro Cytotoxicity Study

Cytotoxicity of the developed and optimized NEs was performed according to the International Organization for Standardization (ISO/EN 10993-5) on mouse embryonic fibroblast (NIH-3T3) cell line, which were procured from American Type Cultural Collection (ATCC). NIH-3T3 was cultured in Dulbecco’s modified Eagle medium (DMEM) with penicillin-streptomycin (1%) and FBS (10%) at 37 °C with a humidified 5% CO_2_/95% air atmosphere. Firstly, the cells (30 × 10^4^) were incubated in a 96 well plate with DMEM for 24 h. Subsequently, cells were incubated with blank NE, drug loaded NE and finalized CNNE (1% CNNE) at the concentration range of 0.25–1.5 µM for 24 h. After incubation, 20 µL of alamarBlue reagent was added to the treated cells followed by 4 h incubation prior to analysis [35]. A microplate reader was used to measure the absorbance of each sample at 570 nm.

Cell viability was calculated as:

(3) $$Cell\;viability\;\left(\%\right)=\;\frac{A_{570}\;of\;treated\;cells}{A_{570}\;of\;control\;cells}\;\times 100$$

### 2.13. Animal Facility

To perform the animal experiment for the wound healing assay, healthy adult female albino Wistar rats of 200–250 g were procured from a registered breeder (Zydus Research Centre (Ahmedabad, Gujarat, India). The animals were retained in the standard conditions of the laboratory (temperature: 25 ± 2 °C and relative humidity: 55 ± 5%) for a period of seven days under 12 h/12 h light and dark cycle with free access of water and food at the animal house of the Institute of Pharmacy, Nirma University, Ahmedabad, India. The study protocol for the current study was approved by the institutional animal ethics committee (IP/PCEU/FAC/25/2019/047, dated; July 20, 2019) according to guidelines mentioned by the Committee for the Purpose of Control and Supervision of Experiments on Animals (CPCSEA), India.

### 2.14. Abrasion Wound Healing Model

The acclimatized animals in the laboratory conditions were anaesthetized by intraperitoneal administration of thiopental (45 mg/kg) [36]. The dorsal hairs of the experimental animals were smooth-shaven, the skin of each animal was disinfected with 70% alcohol and abrasion wound (~1 cm^2^) created with the help of coarse sandpaper and acetone until the skin was bleeding and oozing of fluid was seen, confirming superficial damage of the skin. Then, 50 µL aliquot of bacterial suspension containing Escherichia coli was added to the wound area of each rat. Thereafter the animals were divided into three groups containing six animals in each, where group 1 animals were not treated with any treatment (negative control group), animals in group 2 were treated with blank chitosan-coated NE and the animals in group 3 were treated with formulated CNNE (1%). The treatments (1 mL) were carried out topically to the created wound, at a frequency of once daily for 14 days with complete covering of the wound using sterilized dressing. The animals after creating the wound were kept in different cages to limit any harm by the other animals in the group. Progressive changes of the wounds were recorded by measuring the diameter of the wound using a Vernier caliper every day and the photographs were taken on the 0th day and 14th day using camera [37].

### 2.15. Histopathological Study

The treated animals were again anaesthetized on the 14th day of the experiment and the wound area containing dermis and hypodermis was carefully removed with a sharp scalpel and trimmer for conducting histological analysis. All the skin samples from all groups were preserved in freshly prepared 10% formalin buffer solution. The sections of the wound area were made following preparation of paraffin embedding and block preparation, which were then stained using hematoxylin and eosin (H & E) stain. The sections of skin were examined under microscope to determine the changes. Evaluation of the histological examinations was made to assess the density of blood vessels, thickness of epidermis and number of inflammatory cells [37].

### 2.16. Statistical Analysis

All the experimental data were presented as mean ± standard deviation (SD), where the statistical significance was analyzed using one-way analysis of variance (ANOVA) followed by the Tukey’s test. The significant alterations were designated by *p* < 0.05.

## 3. Results and Discussion

### 3.1. Selection of Suitable Components for the Development of NAR Nanoemulsion

**Figure 2:**
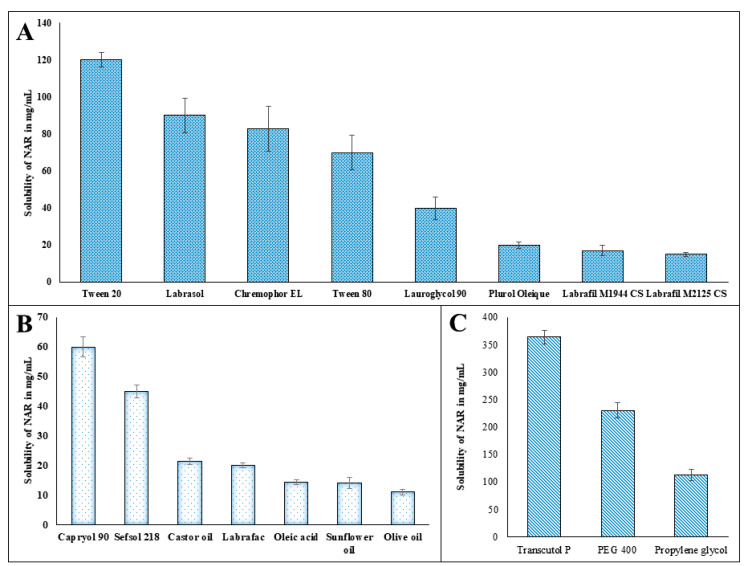
Solubility profile of naringenin (NAR) in different components of nanoemulsion: (**A**) surfactants; (**B**) oils and (**C**) co-surfactants.

The hydrophobic nature of NAR limits its application in pharmaceutical dosage forms. Thus, to overcome the solubility issue of NAR, an NE was formulated where the hydrophobic drug was incorporated within the oil core of the formulation. Furthermore, selection of suitable components for the development of NE was determined in different components. Solubility profiles of NAR in different components were depicted in Figure 2A–C, where maximum solubility of NAR was obtained in Capryol 90 (59.991 ± 3.49 mg/mL) (Figure 2B), Tween 20 (119.99 ± 3.86 mg/mL) (Figure 2A) and Transcutol P (363.72 ± 12.73 mg/mL) (Figure 2C). Therefore, Capryol 90, Tween 20 and Transcutol P were selected as oil, surfactant and cosurfactant for the development of NE in the next stage.

### 3.2. Pseudoternary Phase Diagrams in Determining Optimum Smix Ratio

Penetration of lipophilic fragments of the surfactant molecules into the oil globules is necessary to stabilize the formulated NEs. This increased fluidity at the interface of the two immiscible phases helps in reduction of the interfacial tension. Additionally, the co-surfactants also perform similar role in reduction in the hindrance of such penetration of hydrophobic tails of the surfactants within the oil droplets [38].

**Figure 3:**
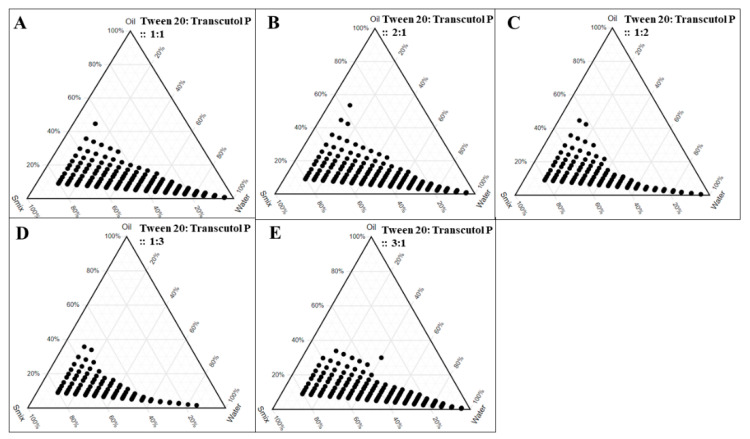
Pseudoternary phase diagrams of Capryol 90 (oil phase) to obtain the nanoemulsion area (as denoted by solid circles) with different ratios of Smix (Tween 20 and Transcutol P): **A**. 1:1, **B**. 2:1, **C**. 1:2, **D**. 1:3, and **E**. 3:1.

The NE regions in the pseudoternary diagrams were represented in Figure 3, where Capryol 90 and varying ratios of oil and Smix were used. From the ternary phase diagrams it is clearly depicted that there is a decreasing concentration of surfactant in the Smix, in other words, from 2:1 (Figure 3B) to 1:3 (Figure 3D), there is gradual decrease in NE region. This could be explained by the fact that decreased concentration of surfactant in the system could not effectively coat the dispersed lipid phase, thereby resulting in a decreased NE region [39]. Furthermore, the transient negative interfacial tension of the oil (Capryol 90) and surfactant (Tween 20) was reduced to its maximum level at the 2:1 ratio of surfactant and co-surfactant, indicating that the resultant mixture is able to increase the fluidity at the interface to form the NE.

### 3.3. Development of NE Formulation

The percentages of oil, Smix and aqueous phase were varied in order to formulate seventeen different batches of formulation as recommended from the Box–Behnken quadratic design. The randomized batches were formulated with 5–15% of Capryol 90 with 30–40% of Smix and remaining aqueous phase. The aqueous titration method was used to formulate the batches, where characterization of the blank NEs was accomplished for globule size, PDI, surface charge and thermodynamic stability. Spontaneous formation of the NE lowers the possibilities of failure in different tests of thermodynamic stability study, thereby there were no consequences of creaming, cracking and phase separation without any significant variation in globule size and PDI of the formulation when compared to the interpretations of the freshly prepared formulations. Finally, the outcomes of the batches were advanced for optimization using Box–Behnken statistical design.

### 3.4. Optimization of Nanoemulsion by Box–Behnken Statistical Design

As the NEs are dispersions of nanometric oil droplets into the continuous aqueous phase, their droplet size, polydispersity range and surface charge play important roles towards stability of the developed formulations [22].

#### 3.4.1. Effect of Independent Variable on Globule Size

Thus understanding the concept of affecting dependent variables by the input on independent variables would give insight, where the statistical outcome of the effect of independent variables on droplet size is represented in Table 2. The obtained *p*-values in Table 2 represent the statistical significance of the influence of two independent variables, oil% (A) and percentage of Smix (B) on droplet size.

In addition, the predicted regression coefficient (*R²*) *value* of 0.7697 is in reasonable agreement with the adjusted *R²* value (0.9669) with a difference of less than 0.2. The predicted R^2^ value of 0.7697 represented 76.97% of the variability in droplet size with the fitted model, whereas the adjusted R^2^ value of 96.69% is appropriate for comparing models with numerous numbers of independent variables. Moreover, desirable values for signal to noise ratio represented by adequate precision should be greater than 4. In this model, an adequate precision value of 23.297, indicated an adequate signal. Hence, this model could be used to navigate the design space. Further, the generated equation in the fitted model during optimization by the QbD technique for globule size is represented below.

Y1 = + 28.35 + 47.66 × A − 32.59 × B + 6.49 × C + 40.79 × A^2^ + 11.94 × A × B − 0.4325 × A × C + 25.92 × B^2^ − 13.55 × B × C + 0.3408 × C^2^ (4)

**Figure 4:**
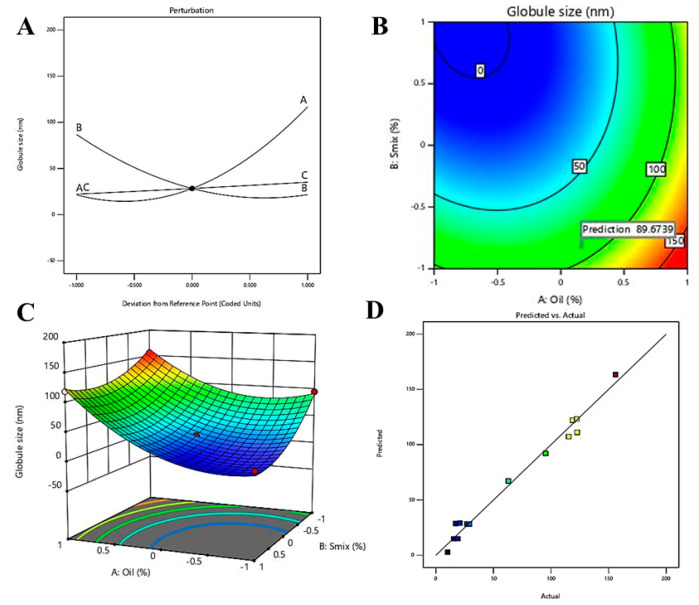
Effect of independent variables on droplet size: (**A**) perturbation graph, (**B**) contour plot, (**C**) 3D surface plot and (**D**) predicted vs. actual graph.

The regression coefficients for A and C are +47.66 and +6.49, respectively, in the equation whereas it is −32.59 for B. Higher value and positive regression coefficient for A along with significant *p*-value (<0.0001) represented significant increase in droplet size along with increase oil% (Table 2). Whereas, a lower positive regression coefficient for water percentage along with an insignificant *p*-value (0.0815) represented an insignificant effect of aqueous phase on droplet size. In contrast, inverse relation between Smix% and droplet size is reflected by negative regression coefficient and significant *p*-value (<0.0001) (Table 2). The closeness of observed and predicted values are represented in Figure 4D.

The perturbation plot, contour plot and 3D surface plot for globule size are represented in Figure 4A–C, respectively, where the relation between independent variables and the dependent variable, globule size, are in agreement with Table 2 and Equation (4). It is clearly depicted that the two independent variables, A (oil%) and B (Smix%), have significant effect on droplet size. As per the perturbation plot and 3D surface plot, oil% have the most significant effect on droplet size, which is further supported by the highest coefficient of A in Equation (4). The increase in droplet size with increase oil% in the formulation might be explained by the fact that increase ratio of oil to Smix is reflected by reduction of interfacial tension between the interface of two immiscible liquids. Additionally, the inverse relation between percentage of Smix and droplet size can be explained by the fact of that increased Smix% helps in reducing interfacial tension between oil and aqueous phase and thereby provides successful coating over the nano droplets which further leads to stabilization of the developed NEs [40]. These findings are in agreement with reported literatures [41,42].

#### 3.4.2. Effect of Independent Variable on PDI

The value of PDI in an NE can be varied from 0 to 1, where values near to 1 are representing more polydispersion, thus, PDI values near to 0 are usually preferred [43]. The ANOVA data for quadratic model on the effect of three independent variables on PDI is represented in Table 3. The significant model term is represented by *p*-values < 0.05, therefore, in this model A, B, A^2^ and C^2^ are significant model terms for PDI.

**Figure 5:**
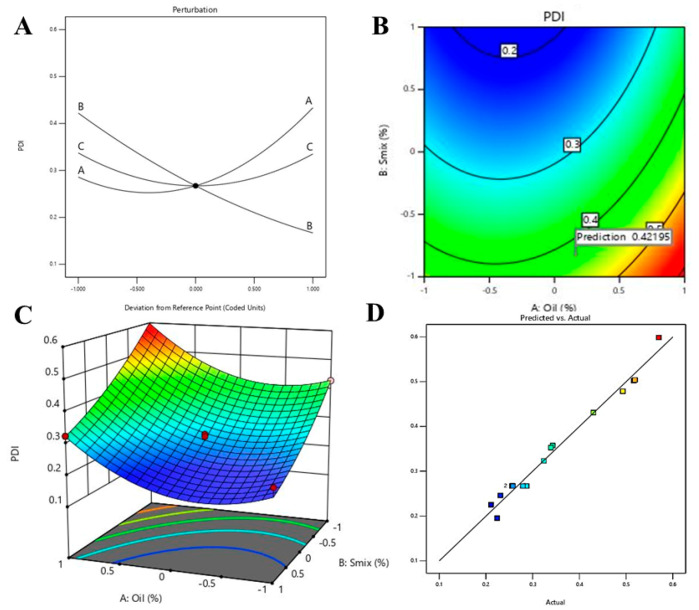
Effect of independent variables on PDI: (**A**) perturbation graph, (**B**) contour plot, (**C**) 3D surface plot and (**D**) predicted vs. actual plot.

In addition to the above explanation, predicted *R² value* (0.7792) is in reasonable agreement with the adjusted *R² value* (0.9611) with less than 0.2 difference. The predicted R^2^ value of 0.7792 represented 77.92% of the variability in PDI with the fitted model, whereas the adjusted R^2^ value of 96.11% is appropriate for comparing models with numerous numbers of independent variables. Moreover, adequate precision (21.597) is more than 4, indicated an adequate signal. Hence, this model could be used to navigate the design space. The closeness of the observed (actual) predicted values for the PDI is represented in Figure 5D. Further, the generated equation during optimization of PDI in the fitted model is represented in the following Equation (5).

Y2 = +0.2672 + 0.0739 × A − 0.1278 × B − 0.0011 × C + 0.0925 × A^2^ − 0.0100 × A × B + 0.0012 × A × C + 0.0273 × B^2^ + 0.0115 × B × C + 0.0690 × C^2^ (5)

Positive regression coefficients of A along with significant *p*-values (<0.0001) represent substantial effect on PDI increment with increase in oil% in the formulation where negative coefficients of B with significant *p*-values (<0.0001) represent significant inverse relation between PDI and percentage of Smix (Table 3).

The perturbation plot, contour plot and 3D surface plot for PDI are presented in Figure 5A–C, respectively, where the relationship between independent variables and PDI are in agreement with Equation (5). As per the perturbation plot, contour plot and 3D surface plot, independent variable B (i.e., Smix%) has the most significant effect on PDI, which is further supported by the highest coefficient of B in Equation (5). The decrease in PDI corresponding to an increase in Smix% might be due to the fact of successful coating of smaller oil droplets using increased concentration of Smix and whereas insufficient Smix concentration may result in coalescence of the droplet and lead to an increase in PDI [26,44]. These findings are in agreement with the previous findings by other researchers [41,42,45].

#### 3.4.3. Effect of Independent Variables on Surface Charge

The technique of zeta potential measurement provides insight into the surface charge of the dispersed globules, which imparts stability of the formulated NEs [46]. The range of zeta potential in the experimental batches of blank NEs was investigated between −8.56 ± 0.12mV and –13.60 ± 0.15 mV; whereas the predicted range was found to be between –8.42 and –13.53 mV, representing closeness of the predicted and actual results. The statistical data on the effect of three independent variables on surface charge is represented in Table 4. Significant model term is represented by *p*-value < 0.05, therefore, in this model B and A^2^ are significant model terms.

**Figure 6:**
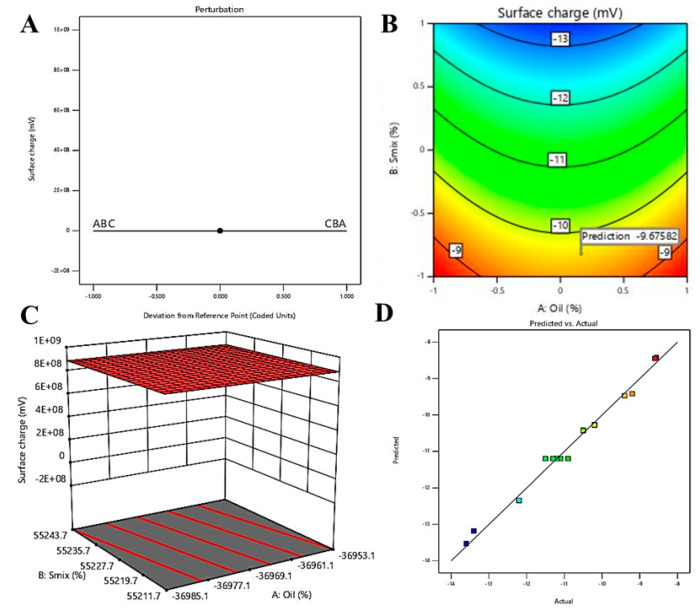
Effect of independent variables on surface charge: (**A**) perturbation graph, (**B**) contour plot, (**C**) 3D surface plot and (**D**) predicted vs. actual.

In addition, predicted *R² value* (0.8927) and adjusted *R² value* (0.9728) is in reasonable agreement with less than 0.2 difference. Moreover, the adequate precision value of 27.416 indicated an adequate signal. Hence, this model could be used to navigate the design space. The close agreement of observed (actual) and predicted values of the surface charge is represented in Figure 6D.

As per the generated quadratic equation (Equation (6)), negative coefficient of three independent variables represent an inverse relation between all three independent variables and surface charge. However, as per Table 4, only B (Smix%) is the significant model term, which is also reflected in Equation (4) by the larger value of regression coefficient compared to coefficient for the other two independent variables.

Y3 = −11.20 − 0.0050 × A − 1.96 × B − 0.0750 × C + 0.0100 × A × B + 0.0000 × A × C − 0.1000 × B × C + 0.9300 × A^2^ − 0.1200 × B^2^ − 0.0800 × C^2^ (6)

The effect of independent variables on the surface charge was depicted using perturbation (Figure 6A), contour plot (Figure 6B) and 3D surface plot (Figure 6C). As depicted, the negative change in surface charge upon increase in concentration of Smix could be explained by the fact of further decrease in surface charge towards negative side. Additionally, Tween 20 being a non-ionic surfactant, presence of alcohol moiety of Transcutol P might be the contributing factor towards the negative zeta potential of the formulations [47].

Following the entire optimization process using this DoE design, a combination of 6% oil, 41% Smix and 53% aqueous phase was designated as the optimized formulation. The particle size of 14.14 ± 0.23 nm and PDI of 0.279 were found for the optimized NE formulation (Supplementary Figure S2). This smaller size and PDI might provide a positive impact towards penetration of the oil globules at the desired site [22]. In the next step, the drug, NAR, was loaded to proceed towards development of desired formulation.

### 3.5. Development of NAR-Loaded NE Formulation

NAR was loaded into the optimized NE formulation by dissolving the drug (to obtain final concentration of 2 mg/mL) in the oil phase and formulated as described above incorporating desired percentages of Smix and aqueous phase. The globule size of the drug loaded NE was determined as 15.69 ± 0.737 nm, whereas the PDI and surface charge were recorded as 0.330 and –8.33 ± 3.09 mV (Supplementary Figure S3).

**Figure 7:**
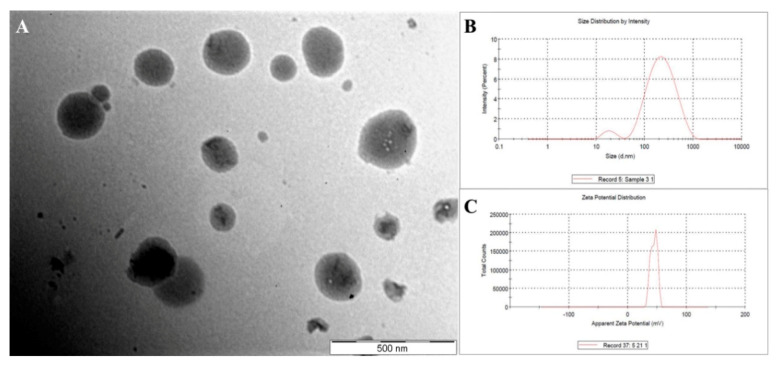
Representation of morphology of the optimized (1%) chitosan-coated naringenin nanoemulsion (**A**), particle size distribution (**B**) and zeta potential (**C**).

The chitosan-coated drug-loaded NEs were formulated using three different concentrations of chitosan solution, following which the formulations were characterized for their change in globule size, PDI and surface charge. Increase in percentage of chitosan in the formulation had been reported to increase the size of the dispersed globules due to formation of coating over the globule; however, lower concentration of chitosan could not form a proper coating [27,48]. Similarly, the globule size of the dispersed globules of the formulated CNNEs was found to be increasing at a chitosan-concentration dependent manner (Table 5 and Figure 7B). On the other hand, the surface charge of the dispersed globules was changed from negative to positive, which is because of coating of the dispersed droplets by the chitosan layer (Table 5 and Figure 7C).

Thus, it could be hypothesized that the positively charged water soluble form of chitosan contains the amine groups at their surface. Thereby, this positive charge facilitates binding to the negatively charged droplet surface, to form the stable coating. Further, the TEM analysis of the 1% CNNE formulation demonstrated the spherical morphology of the coated droplets (Figure 7A). The histogram observed in Figure 7B indicates that the particles in the CNNE formulation possess various sizes, which also substantiate the large PDI value (0.419) observed in Table 5 and the morphological observation in the TEM image (Figure 7A). Additionally, the uniformity of the droplet size (at <200 nm range) without any crystalline NAR confirmed complete entrapment of the drug loaded within the oil phase of the formulation.

### 3.6. Determination of pH and Viscosity of the NE Formulations

pH of the topical formulation plays an important role when applied to the wound environment. The acidic environment of the wound promotes healing, where it is known to modify protease activity, oxygen releasing potential, enhancing epithelization of the cells and angiogenesis including antimicrobial activity [49]. Due to the chronic condition of wounds, the pH is known to be elevated towards the alkaline side, changing from the normal acidic pH of 4.2–5.6 to 6.5–8.5, a condition that is unfavorable for healing [50,51]. The results of the pH measurements were presented in Table 6, which depicted that the pH of the formulations were shifted towards acidic side. Thus, a slightly acidic pH of the developed formulations could be helpful in the healing process of t topical wounds.

Concurrently, optimization of NE viscosity plays an important role as it provides enhancement in residence time of the formulation through imparting a sustained release property to the formulation. Furthermore, for smooth topical application and higher retention at the wound site, an optimum viscosity is desired. Viscosity of the drug-loaded NE was not significantly different from the blank NE (52.33 ± 1.25 cP) (*p* < 0.01). However, presence of chitosan coating leads to a noticeable concentration-dependent increment of viscosity (Table 6). This increased viscosity of the CNNEs is expected to provide increased retention of the formulation at the wound site with controlled release of entrapped medication to obtain its therapeutic efficacy.

### 3.7. Ex Vivo Mucoadhesive Strength

**Figure 8:**
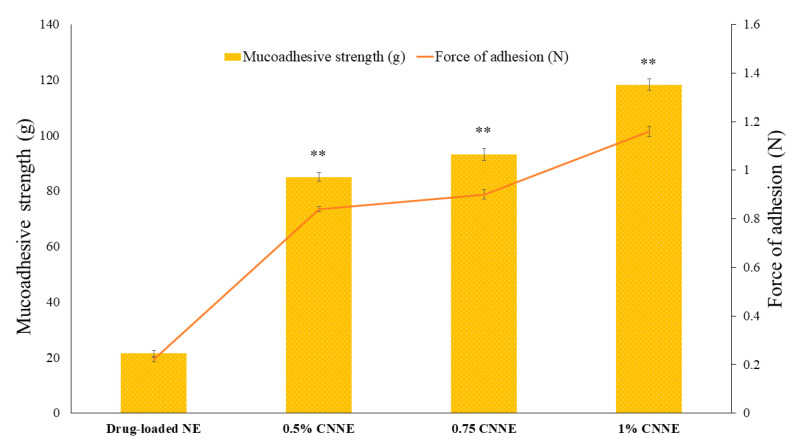
Mucoadhesion strength and force of adhesion of different nanoemulsions measured by the mucoadhesive strength test. Values are expressed as mean ± SD (*n* = 3), where ** represents significant difference from blank nanoemulsion (*p* < 0.01).

Mucoadhesion is an important parameter for retention of formulation at the site of action when applied externally. As a predictor of mucoadhesive property, mucoadhesive strength and force of adhesion was measured which is the indicator of maximum force required for detachment of goat skin from the formulations. As illustrated in Figure 8, force of adhesion for CNNEs was found to be significantly higher than NAR-loaded NEs (*p* < 0.01). However, no difference was observed between blank and drug-loaded NE, which displayed the non-adhesive nature of the drug (Supplementary Table S1). Additionally, the mucoadhesive property of the CNNEs was found to be increased with increment in chitosan concentration within the formulation. This increment in mucoadhesion in the presence of chitosan is attributed to the functional group present in chitosan that allows interaction with the skin [21,52]. It is also reported that chitosan is a cationic polymer, having positive charge on the surface, which enhances the interaction with negatively charged stratum corneum. This interaction leads to change in the morphology of the epidermis outer layer by breaking the tight conjugation of corneocyte layers. It depicted the permeation enhanced behavior which tends to enhance transport of hydrophobic drug [52]. Therefore, it is expected that application of the chitosan-coated formulations will be retained at the site of application for longer durations as desired due to their mucoadhesive property to supply the entrapped wound healing components.

### 3.8. In Vitro Drug Release of NE

**Figure 9:**
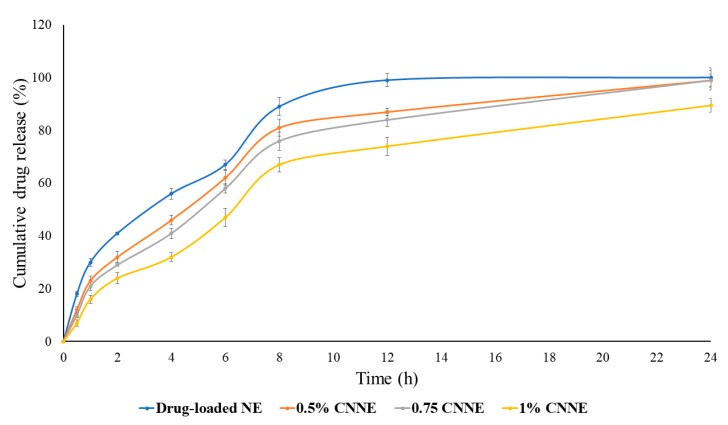
*In vitro* release profile of drug-loaded NE and chitosan coated NE formulations.

An in vitro release study was carried out to compare the release pattern of the drug from NAR-loaded NE and CNNEs. Figure 9 illustrates the cumulative release of NAR, where the higher released profile of NAR was noticed from the NE formulation compared to the CNNEs. Total (100.0%) release of NAR was observed from the NE within 12 h of the experimental period; however, 0.5% CNNE, 0.75% CNNE and 1% CNNE showed 99.0%, 99.2% and 89.5% release of NAR, respectively (Figure 9). No drug precipitation was observed during release study as confirmed by the physical observation of the dialysis bag. The agenda of chitosan-coating of the NE is to control burst release phenomenon by NE as well as maintain the sustained duration of release. Moreover, 0.5% and 0.75% did not provide gradual initial release as desired but coating with 1% produced desired sustained release with low level of burst release. These results are coherent with viscosity and mucoadhesive strength. The release of hydrophobic drug form NE is dependent on the diffusion ability from the oil core into surfactant layer and finally to continuous phase. Regarding CNNEs, the dynamic layer of chitosan creates additional partition through continuous phase. This leads to slow release of drug from the oil core and it could be predicted that it will maintain the therapeutic window at the site of action for a longer period of time [53]. Therefore, it is expected to obtain a prolonged release of incorporated NAR at the site of application to maintain the supply of the therapeutic agent towards effective healing of wound.

Concurrently, the release data was fitted into various release kinetic models to identify release mechanisms of NAR from the formulated NEs. Drug release kinetic data reveals that CNNEs showed zero order release pattern. In contrast, drug-loaded NE displayed a first order release pattern. However, all the formulations demonstrated Higuchi release mechanism, which represents release of drug by diffusion mechanism (Table 7).

Based on the above findings on CNNEs, 1% CNNE was selected for further study. The size of the dispersed globules in the 1% CNNE was around 150 nm, however, the viscosity, and subsequent mucoadhesive property would allow the formulation to persist at the site of application for a longer duration. Additionally, the slowest zero-order release profile through Higuchi model might allow the formulation to act longer at the site of application.

### 3.9. In Vitro Cytotoxicity Study

**Figure 10:**
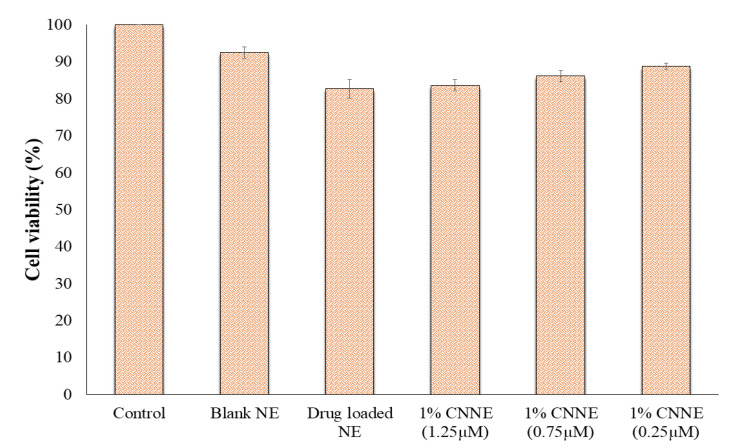
Cell viability analysis on embryonic fibroblast (NIH-3T3) cells upon treatment of blank NE, drug-loaded NE and different concentrations of CNNE (1%) at 24 h. The cell viability was determined by in vitro cytotoxicity assay. The control consisted of cells treated with 0.1% dimethyl sulfoxide (vehicle control representing 100% cell viability). Data are expressed as mean ± SD (*n* = 3).

Considering evaluation of biocompatibility of the pharmaceutical formulation, cytotoxicity is the first parameter. According to ISO-10993, cytocompatibility evaluation of material and medical devices can be done based on cell count, cellular activity and morphology. The NIH-3T3 mouse fibroblast cells had been used to evaluate cytotoxicity of different pharmaceuticals as fibroblast cells are the main part of connective tissue and matrix of the body, thus widely adopted for topical formulation studies to check cell viability and genotoxicity [37,54]. Cytotoxicity of NE was evaluated by direct contact assay in which test material was placed directly onto the cell medium to affect the cell viability. Dose-dependent cytotoxicity assay results revealed that blank NE have the least cytotoxicity (92.45%), possessing the biocompatibility of the excipient used in our formulation. However, CNNE (1%) (1.25 µM) showed insignificant toxicity (82.65%) compared to blank NE. As evident from literature, NAR have prominent cytotoxic and apoptotic effects on cancer cells in a dose-dependent manner due to increased reactive oxygen species (ROS) generation. Significant negative relationships between cell viability and ROS are observed (*p* < 0.001) by Kocyigit et al. [55]. On the other hand, remarkably lower cytotoxicity was reported on normal cell lines [55,56]. On the other hand, presence of chitosan enhances the cytocompatibility in a dose dependent manner compared to drug-loaded NE. As illustrated in Figure 10, 1% CNNE at the dose of 0.25, 0.75 and 1.25 µM showed cell viability of 88.74%, 86.12% and 83.56%, respectively. While comparing the cell viability of NAR-loaded NE and 1% CNNE in the same dose, it was indicated that the presence of chitosan does not induce any significant changes in cell growth. However, a slight increment is due to slow release of NAR from the formulation. 

### 3.10. Construction of Wound Healing

Construction of wound following abrasion and adding of bacterial suspension is an established model, where the bacterial contamination delays in the healing process of the animals. Due to contamination, there will be a competition for nutrients and oxygen between the invading bacteria and host resistance cells (macrophages and fibroblasts), and thereby the wound healing process is delayed [57]. Therefore, application of our optimized formulation can rapidly reduce the diameter of the created wounds if it possesses favorable wound healing characteristics as the changes of the wound diameter is an obvious process of normal wound healing method.

**Figure 11:**
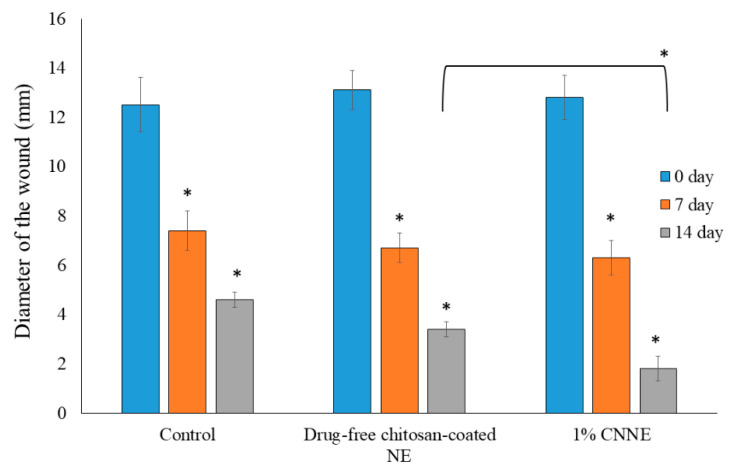
Representation of wound constructions in different experimental groups on the macroscopic measurements. Data are presented as mean ± SEM, where * *p* < 0.05 compared to the control group (*n* = 6).

**Figure 12:**
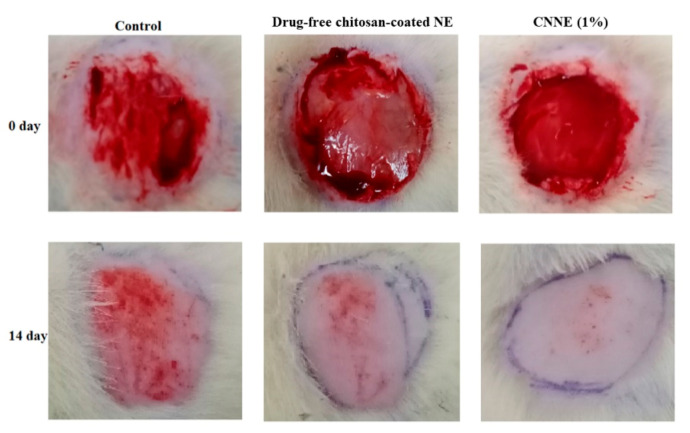
Representative photographic images of wound areas in control, drug-free chitosan-coated NE formulation, and CNNE (1%) treated Wistar rats on days 0 and 14.

The observed readings on reduction in wound diameter within the two weeks of the experimental period were depicted in Figure 11. Furthermore, the pictorial differentiation of the wound conditions at 0-day and 14th day for the treated and untreated animals was presented in Figure 12. There is a significant reduction in wound diameter observed on subsequent results when compared to the 0-day data in each groups; however, when the 14th day data of untreated group was compared to the CNNE treated animals, it showed a significant reduction in the wound healing. The wound healing efficacy by the drug-free NE could be explained by the antibacterial and wound healing potential of chitosan, whereas significant increase in CNNE treated results on the 14th day of experiment might be explained by the synergistic effect of NAR and chitosan on wound healing [58]. Moreover, functional and rapid closure of the wound is always a principal target when aiming for wound therapy. The results represented above depicted a progressive healing of the created wound in the experimental animals at a faster rate, suggesting a constructive effect of the developed formulation.

### 3.11. Histopathological Assay

**Figure 13:**
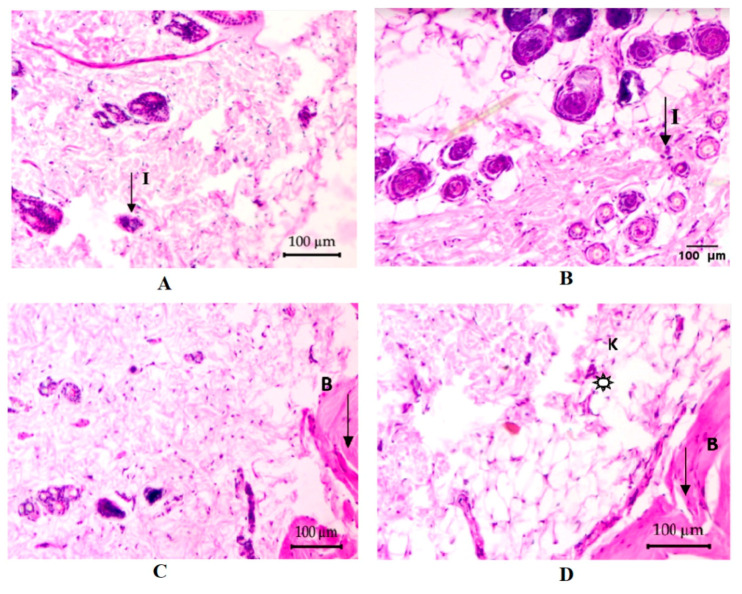
Representative photomicrographs of the rat skin tissues in the control, drug-free chitosan-coated NE formulation and CNNE (1%) treated groups of abrasion model in albino Wistar rats. (**A**) Wound area before the treatment at day 0, (**B**) control group at day 14, (**C**) drug-free chitosan-coated NE formulation treated group at day 14 and (**D**) CNNE (1%) treated group at day 14. (I: inflammatory cells; B: blood vessels; K: keratinization; 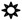: granulated tissue).

The microscopic examination of the tissues aids in diagnosing the condition of the cells pertaining to management of the patient. The photomicrographs (Figure 13A, 0 day) of rat tissue sections (100 µm/100×) stained with H & E correspond to the negative control rat skin showing global granulation tissue formation and also having infiltration of inflammatory cells as active inflammatory response. Upon completion of the 14-day treatment period of the negative control treatment group, Figure 13B is showing a worsening pathology of increased global granulation tissue formation in dermis as active inflammatory response. In addition, subcutaneous tissue is showing focal suppuration, defected or changed normal flora to dermis. Alternatively, treatment of the second group animals with the blank formulation for 14 days is showing acanthosis into the dermis and infiltration of inflammatory cells into it (Figure 13C). Mild to moderate hyperkeratosis is also shown in the dermis. On the other hand, CNNE (1%) treated animals, after 14 days, are showing improved morphology with mild to moderate penetration of inflammatory cells infiltration into the dermis (Figure 13D). Overall, the migration of cells for effective healing of the wounds was found to be faster in CNNE (1%) treated animals compared to the control, and blank formulation treated animals, suggesting superiority of our formulation approach towards wound healing.

In continuation of the previous discussion, we would like to emphasize that the topical application of NAR stimulates anti-inflammatory and antioxidant effects, thereby known to show significant increase in wound contraction [59]. Reported antioxidant and angiogenic roles of NAR also potentiate healing of wounds through restraining endothelial apoptosis through growth factor and inflammatory mediator modulation [60]. Thus, comparison of different inflammatory parameters in Table 8 shows that there is an increase in numbers of blood vessels in the treatment groups following 14 days; however, the angiogenic potential is high when the wounds are treated with the developed CNNE. Furthermore, the thickness of the wounded skin of CNNE treated animals was decreased because of a decrease in migration of inflammatory cells. It has been established that there is always an influx of inflammatory cells to the site of wounds creating an inflammatory condition, thus the reading on day 0 (Table 8), shows that there was high concentration of inflammatory cells. This influx of neutrophils is known to generate free radicals at the site of wounds; however, due to the antioxidant potential of NAR, there might be neutralization of the generated radicals and there is termination of the inflammatory phase and progression towards the proliferative stage for faster healing of the wound [60].

## 4. Conclusions

In summary, a suitable chitosan-coated NAR loaded NE formulation was developed by the use of the Box–Behnken statistical design, where the size of the dispersed chitosan coated oil droplets were found to ~150 nm. The positive surface charge of the formulated CNNE could potentiate stability of the formulation through repulsive interaction between the dispersed globules. Significant increase in the mucoadhesive strength of the CNNE (1%) will possess retention of the formulation at the wound site for longer duration, whereas sustained release of the drug will maintain the drug concentration for prolonged healing of the wound. Finally, low cytotoxicity in fibroblast cells and accelerated wound healing potential of the CNNE (1%) formulation reflecting positive effects of NAR and chitosan towards wound healing. Thus, the antioxidant, antimicrobial, anti-inflammatory and angiogenic properties of the phytoconstituents in the formulation might be responsible for the synergistic response of the formulation towards effective healing of the wound. Therefore, it could be said that chitosan-based nanoemulgel formulations containing NAR would provide a novel perception towards treatment of chronic wounds with new hope, to reduce the global burden in the near future. However, extensive studies are necessary to establish the efficacy and safety of the formulation further in different preclinical and clinical studies.

## Figures and Tables

**Table 1:** Box–Behnken statistical design: The generated experimental runs along with independent variables at their three levels and experimental dependent variables for blank nanoemulsions (NEs).

**Batch**	**Values of Independent Variables**			**Actual Responses**		
	**A (% *v/v*)**	**B (% *v/v*)**	**C (% *v/v*)**	**Y1 (nm)**	**Y2**	**Y3 (mV)**
1	0	−1	1	115.39	0.493	−9.2
2	0	1	1	19.1	0.231	−13.6
3	1	−1	0	156	0.57	−8.6
4	0	0	0	29.29	0.258	−11.3
5	1	1	0	118.7	0.324	−12.2
6	−1	−1	0	95.3	0.43	−8.56
7	−1	0	−1	15.69	0.343	−10.2
8	0	0	0	29.14	0.279	−11.5
9	0	−1	−1	63.01	0.519	−9.4
10	1	0	1	122.4	0.517	−10.5
11	0	0	0	26.98	0.256	−11.2
12	0	0	0	27.45	0.287	−10.9
13	1	0	−1	122.6	0.516	−10.2
14	0	0	0	28.87	0.256	−11.1
15	0	1	−1	20.93	0.211	−13.4
16	−1	1	0	10.23	0.224	−12.2
17	−1	0	1	17.22	0.339	−10.5
**Independent Variable**	**Levels**					
	**Low** **(−1)**		**Medium** **(0)**		**High** **(+1)**	
A = Oil (%*v/v*)	5%		10%		15%	
B = Smix (%*v/v*)	35%		40%		45%	
C = Water (%*v/v*)	40%		50%		60%	
**Dependent variables**						
Y1 = Particle size						
Y2 = Polydispersity index						
Y3 = Zeta potential						

**Table 2:** Analysis of variance data for droplet size.

Source	Sum of Squares	Degree of freedom (Df)	F-Ratio	*p*-Value
A: Oil	18,169.90	1	223.31	<0.0001
B: Smix	8498.17	1	104.44	<0.0001
C: Aqueous phase	336.44	1	4.13	0.0815
A^2^	7005.83	1	86.10	<0.0001
AB	570.49	1	7.01	0.0330
AC	0.7482	1	0.0092	0.9263
BC	734.68	1	9.03	0.0198
B^2^	2828.99	1	34.77	0.0006
C^2^	0.4889	1	0.0060	0.9404

**Table 3:** Analysis of variance data for polydispersity index (PDI).

Source	Sum of Squares	Df	F-Ratio	*p*-Value
A: Oil	0.0437	1	73.67	<0.0001
B: Smix	0.1306	1	220.30	<0.0001
C: Aqueous phase	0.0000	1	0.0171	0.8997
A^2^	0.0360	1	60.82	0.0001
AB	0.0004	1	0.6749	0.4384
AC	6.250 × 10^−6^	1	0.0105	0.9211
BC	0.0005	1	0.8926	0.3762
B^2^	0.0031	1	5.29	0.0551
C^2^	0.0201	1	33.85	0.0007

**Table 4:** Analysis of variance data for surface charge.

Source	Sum of Squares	Df	F-Ratio	*p*-Value
A: Oil	0.0002	1	0.0034	0.9552
B: Smix	30.58	1	517.74	<0.0001
C: Aqueous phase	0.0450	1	0.7620	0.4117
A^2^	3.64	1	61.66	0.0001
AB	0.0004	1	0.0068	0.9367
AC	0.0000	1	0.0000	1.0000
BC	0.0400	1	0.6773	0.4376
B^2^	0.0606	1	1.03	0.3447
C^2^	0.0269	1	0.4563	0.5210

**Table 5:** Analysis of variance data for surface charge.

Formulation Details	Globule Size (nm)	PDI	Zeta Potential (mV)
NAR-loaded NE	15.69 ± 2.32	0.330	–8.33 ± 3.09
0.5% chitosan-coated naringenin nanoemulsion (CNNE)	97.48 ± 6.23	0.245	+29.5 ± 6.40
0.75% CNNE	105.3 ± 6.56	0.222	+33.8 ± 3.30
1% CNNE	156 ± 7.34	0.419	+44.4 ± 5.57

**Table 6:** Representation of pH and viscosity of the nanoemulsion (NE) formulations.

Formulation Details	Measured pH	Measured Viscosity (cP)
NAR-loaded NE	5.84 ± 0.060	54.66 ± 1.52
0.5% CNNE	4.34 ± 0.060	169.3 ± 5.13
0.75% CNNE	4.44 ± 0.045	335 ± 8.55
1% CNNE	4.53 ± 0.150	658.3 ± 12.08

**Table 7:** Representation of pH and viscosity of the NE formulations.

Formulation	Zero-Order	First Order	Higuchi	Hixon–Crowell
Drug-loaded NE	0.956	0.867	0.968	0.893
0.5% CNNE	0.972	0.886	0.981	0.923
0.75% CNNE	0.966	0.879	0.975	0.931
1.0% CNNE	0.976	0.865	0.951	0.910

**Table 8:** Comparative representation of histopathological data for different treatment groups.

Parameters	Control		Drug-Free Chitosan-Coated NE		CNNE (1%)	
	0 Day	14 Day	0 Day	14 Day	0 Day	14 Day
Density of blood vessels	+	++	+	++	+	+++
Thickness of epidermis	+	+	+	++	+	+++
Number of inflammatory cells	+++	++	+++	++	+++	+

(+) low, (++) medium, and (+++) high.

## Supplementary Materials

The following are available online at https://www.mdpi.com/1999-4923/12/9/893/s1: Figure S1: Calibration curve for determination of unknown concentration of naringenin using UV-visible spectrophotometer, Figure S2: Representation of blank nanoemulsion particle size distribution, Figure S3: Representation of naringenin-loaded nanoemulsion particle size distribution and zeta potential, Table S1: Representation of mucoadhesive strength and force of adhesion of the formulated formulations on goatskin.

## References

[1] Croitoru A.-M., Ficai D., Ficai A., Mihailescu N., Andronescu E., Turculet C.F. (2020). Nanostructured Fibers Containing Natural or Synthetic Bioactive Compounds in Wound Dressing Applications. Materials (Basel).

[2] Morsy M.A., Abdel-Latif R.G., Nair A.B., Venugopala K.N., Ahmed A.F., Elsewedy H.S., Shehata T.M. (2019). Preparation and Evaluation of Atorvastatin-Loaded Nanoemulgel on Wound-Healing Efficacy. Pharmaceutics.

[3] Moroz A., Deffune E. (2013). Platelet-rich plasma and chronic wounds: Remaining fibronectin may influence matrix remodeling and regeneration success. Cytotherapy.

[4] Morton L.M., Phillips T.J. (2016). Wound healing and treating wounds: Differential diagnosis and evaluation of chronic wounds. J. Am. Acad. Dermatol..

[5] Gould L., Abadir P., Brem H., Carter M., Conner-Kerr T., Davidson J., DiPietro L., Falanga V., Fife C., Gardner S. (2015). Chronic wound repair and healing in older adults: Current status and future research. J. Am. Geriatr. Soc..

[6] Tottoli E.M., Dorati R., Genta I., Chiesa E., Pisani S., Conti B. (2020). Skin Wound Healing Process and New Emerging Technologies for Skin Wound Care and Regeneration. Pharmaceutics.

[7] Cowin A.J. (2019). New Innovations in Wound Healing and Repair. Int. J. Mol. Sci..

[8] Choudhury H., Pandey M., Lim Y.Q., Low C.Y., Lee C.T., Marilyn T.C.L., Loh H.S., Lim Y.P., Lee C.F., Bhattamishra S.K. (2020). Silver nanoparticles: Advanced and promising technology in diabetic wound therapy. Mater. Sci. Eng. C.

[9] Matica M.A., Aachmann F.L., Tøndervik A., Sletta H., Ostafe V. (2019). Chitosan as a Wound Dressing Starting Material: Antimicrobial Properties and Mode of Action. Int. J. Mol. Sci..

[10] Dehkordi A.N., Babaheydari F.M., Chehelgerdi M., Dehkordi S.R. (2019). Skin tissue engineering: Wound healing based on stem-cell-based therapeutic strategies. Stem Cell Res. Ther..

[11] Sivamani R.K., Ma B.R., Wehrli L.N., Maverakis E. (2012). Phytochemicals and Naturally Derived Substances for Wound Healing. Adv. Wound Care.

[12] Salehi B., Fokou P.V.T., Sharifi-Rad M., Zucca P., Pezzani R., Martins N., Sharifi-Rad J. (2019). The Therapeutic Potential of Naringenin: A Review of Clinical Trials. Pharmaceuticals (Basel).

[13] Den Hartogh D.J., Tsiani E. (2019). Antidiabetic Properties of Naringenin: A Citrus Fruit Polyphenol. Biomolecules.

[14] Md S., Alhakamy N.A., Aldawsari H.M., Husain M., Kotta S., Abdullah S.T., Fahmy U.A., Alfaleh M.A., Asfour H.Z. (2020). Formulation Design, Statistical Optimization, and In Vitro Evaluation of a Naringenin Nanoemulsion to Enhance Apoptotic Activity in A549 Lung Cancer Cells. Pharmaceuticals (Basel).

[15] Parashar P., Rathor M., Dwivedi M., Saraf S.A. (2018). Hyaluronic Acid Decorated Naringenin Nanoparticles: Appraisal of Chemopreventive and Curative Potential for Lung Cancer. Pharmaceutics.

[16] Jacob S., Nair A.B. (2018). Cyclodextrin complexes: Perspective from drug delivery and formulation. Drug Dev. Res..

[17] Islam N., Dmour I., Taha M.O. (2019). Degradability of chitosan micro/nanoparticles for pulmonary drug delivery. Heliyon.

[18] Elieh-Ali-Komi D., Hamblin M.R. (2016). Chitin and Chitosan: Production and Application of Versatile Biomedical Nanomaterials. Int. J. Adv. Res. (Indore).

[19] Ways T.M.M., Lau W.M., Khutoryanskiy V.V. (2018). Chitosan and Its Derivatives for Application in Mucoadhesive Drug Delivery Systems. Polymers.

[20] Kumria R., Al-Dhubiab B.E., Shah J., Nair A.B. (2018). Formulation and Evaluation of Chitosan-Based Buccal Bioadhesive Films of Zolmitriptan. J. Pharm. Innov..

[21] Liu H., Wang C., Li C., Qin Y., Wang Z., Yang F., Li Z., Wang J. (2018). A functional chitosan-based hydrogel as a wound dressing and drug delivery system in the treatment of wound healing. RSC Adv..

[22] Choudhury H., Gorain B., Chatterjee B., Mandal U.K., Sengupta P., Tekade R.K. (2017). Pharmacokinetic and Pharmacodynamic Features of Nanoemulsion Following Oral, Intravenous, Topical and Nasal Route. Curr. Pharm. Des..

[23] Gorain B., Choudhury H., Nair A.B., Dubey S.K., Kesharwani P. (2020). Theranostic application of nanoemulsions in chemotherapy. Drug Discov. Today.

[24] Choudhury H., Gorain B., Pandey M., Chatterjee L.A., Sengupta P., Das A., Molugulu N., Kesharwani P. (2017). Recent Update on Nanoemulgel as Topical Drug Delivery System. J. Pharm. Sci..

[25] Kumbhar S.A., Kokare C.R., Shrivastava B., Choudhury H. (2020). Specific and Sensitive RP-HPLC Method Development and Validation for the Determination of Aripiprazole: Application in Preformulation Screening of Nanoemulsion. Cur. Nanomed. (Former. Recent Pat. Nanomed.).

[26] Kumbhar S.A., Kokare C.R., Shrivastava B., Gorain B., Choudhury H. (2020). Preparation, characterization, and optimization of asenapine maleate mucoadhesive nanoemulsion using Box-Behnken design: In vitro and in vivo studies for brain targeting. Int. J. Pharm..

[27] Choudhury H., Zakaria N.F.B., Tilang P.A.B., Tzeyung A.S., Pandey M., Chatterjee B., Alhakamy N.A., Bhattamishra S.K., Kesharwani P., Gorain B. (2019). Formulation development and evaluation of rotigotine mucoadhesive nanoemulsion for intranasal delivery. J. Drug Deliv. Sci. Technol..

[28] Al-Dhubiab B.E., Nair A.B., Kumria R., Attimarad M., Harsha S. (2016). Development and evaluation of buccal films impregnated with selegiline-loaded nanospheres. Drug Deliv..

[29] Gorain B., Choudhury H., Kundu A., Sarkar L., Karmakar S., Jaisankar P., Pal T.K. (2014). Nanoemulsion strategy for olmesartan medoxomil improves oral absorption and extended antihypertensive activity in hypertensive rats. Colloids Interfaces B Biointerfaces.

[30] Shah J., Nair A.B., Jacob S., Patel R.K., Shah H., Shehata T.M., Morsy M.A. (2019). Nanoemulsion Based Vehicle for Effective Ocular Delivery of Moxifloxacin Using Experimental Design and Pharmacokinetic Study in Rabbits. Pharmaceutics.

[31] Pathan I.B., Dwivedi R., Ambekar W. (2019). Formulation and evaluation of ketoprofen loaded chitosan nanogel for pain management: Ex-vivo and In-vivo study. Ars Pharm..

[32] Nair A.B., Shah J., Al-Dhubiab B.E., Patel S.S., Morsy M.A., Patel V., Chavda V., Jacob S., Sreeharsha N., Shinu P. (2019). Development of Asialoglycoprotein Receptor-Targeted Nanoparticles for Selective Delivery of Gemcitabine to Hepatocellular Carcinoma. Molecules (Basel).

[33] Pendekal M.S., Tegginamat P.K. (2012). Formulation and evaluation of a bioadhesive patch for buccal delivery of tizanidine. Acta Pharm. Sin. B.

[34] Jacob S., Nair A.B., Al-Dhubiab B.E. (2017). Preparation and evaluation of niosome gel containing acyclovir for enhanced dermal deposition. J. Liposome Res..

[35] Raja M.A.G., Katas H., Hamid Z.A. (2016). Penetration and silencing activity of VEGF dicer substrate siRNA vectorized by chitosan nanoparticles in monolayer culture and a solid tumor model in vitro for potential application in tumor therapy. J. Nanomater..

[36] Nair A., Morsy M.A., Jacob S. (2018). Dose translation between laboratory animals and human in preclinical and clinical phases of drug development. Drug Dev. Res..

[37] Bektas N., Şenel B., Yenilmez E., Özatik O., Arslan R. (2020). Evaluation of wound healing effect of chitosan-based gel formulation containing vitexin. Saudi Pharm. J..

[38] Pavoni L., Perinelli D.R., Bonacucina G., Cespi M., Palmieri G.F. (2020). An Overview of Micro- and Nanoemulsions as Vehicles for Essential Oils: Formulation, Preparation and Stability. Nanomaterials (Basel).

[39] Azeem A., Rizwan M., Ahmad F.J., Iqbal Z., Khar R.K., Aqil M., Talegaonkar S. (2009). Nanoemulsion components screening and selection: A technical note. AAPS PharmSciTech.

[40] Shah H., Nair A.B., Shah J., Bharadia P., Al-Dhubiab B.E. (2019). Proniosomal gel for transdermal delivery of lornoxicam: Optimization using factorial design and in vivo evaluation in rats. Daru J. Fac. Pharm. Tehran Univ. Med. Sci..

[41] Hidajat M.J., Jo W., Kim H., Noh J. (2020). Effective Droplet Size Reduction and Excellent Stability of Limonene Nanoemulsion Formed by High-Pressure Homogenizer. Colloids Interfaces.

[42] Choudhury H., Gorain B., Karmakar S., Biswas E., Dey G., Barik R., Mandal M., Pal T.K. (2014). Improvement of cellular uptake, in vitro antitumor activity and sustained release profile with increased bioavailability from a nanoemulsion platform. Int. J. Pharm..

[43] Shah J., Nair A.B., Shah H., Jacob S., Shehata T.M., Morsy M.A. (2019). Enhancement in antinociceptive and anti-inflammatory effects of tramadol by transdermal proniosome gel. Asian J. Pharm. Sci..

[44] Chuacharoen T., Prasongsuk S., Sabliov C.M. (2019). Effect of Surfactant Concentrations on Physicochemical Properties and Functionality of Curcumin Nanoemulsions under Conditions Relevant to Commercial Utilization. Molecules (Basel).

[45] Gadhave D., Gorain B., Tagalpallewar A., Kokare C. (2019). Intranasal teriflunomide microemulsion: An improved chemotherapeutic approach in glioblastoma. J. Drug Deliv. Sci. Technol..

[46] Pandey M., Choudhury H., Yeun O.C., Yin H.M., Lynn T.W., Tine C.L.Y., Wi N.S., Yen K.C.C., Phing C.S., Kesharwani P. (2018). Perspectives of Nanoemulsion Strategies in The Improvement of Oral, Parenteral and Transdermal Chemotherapy. Curr. Pharm. Biotechnol..

[47] Ali H.H., Hussein A.A. (2017). Oral nanoemulsions of candesartan cilexetil: Formulation, characterization and in vitro drug release studies. AAPS Open.

[48] Luesakul U., Puthong S., Sansanaphongpricha K., Muangsin N. (2020). Quaternized chitosan-coated nanoemulsions: A novel platform for improving the stability, anti-inflammatory, anti-cancer and transdermal properties of Plai extract. Carbohydr. Polym..

[49] Kuo S.-H., Shen C.-J., Shen C.-F., Cheng C.-M. (2020). Role of pH Value in Clinically Relevant Diagnosis. Diagnostics (Basel).

[50] Ono S., Imai R., Ida Y., Shibata D., Komiya T., Matsumura H. (2015). Increased wound pH as an indicator of local wound infection in second degree burns. Burns.

[51] Nair A., Jacob S., Al-Dhubiab B., Attimarad M., Harsha S. (2013). Basic considerations in the dermatokinetics of topical formulations. Braz. J. Pharm. Sci..

[52] Tan Q., Liu W., Guo C., Zhai G. (2011). Preparation and evaluation of quercetin-loaded lecithin-chitosan nanoparticles for topical delivery. Int. J. Nanomed..

[53] Natesan S., Sugumaran A., Ponnusamy C., Thiagarajan V., Palanichamy R., Kandasamy R. (2017). Chitosan stabilized camptothecin nanoemulsions: Development, evaluation and biodistribution in preclinical breast cancer animal mode. Int. J. Biol. Macromol..

[54] Abdel-Rahman R.M., Abdel-Mohsen A.M., Hrdina R., Burgert L., Fohlerova Z., Pavliňák D., Sayed O.N., Jancar J. (2016). Wound dressing based on chitosan/hyaluronan/nonwoven fabrics: Preparation, characterization and medical applications. Int. J. Biol. Macromol..

[55] Kocyigit A., Koyuncu I., Dikilitas M., Bahadori F., Turkkan B. (2016). Cytotoxic, genotoxic and apoptotic effects of naringenin-oxime relative to naringenin on normal and cancer cell lines. Asian Pac. J. Trop. Biomed..

[56] Kumar S.P., Birundha K., Kaveri K., Devi K.T.R. (2015). Antioxidant studies of chitosan nanoparticles containing naringenin and their cytotoxicity effects in lung cancer cells. Int. J. Biol. Macromol..

[57] Dai T., Kharkwal G.B., Tanaka M., Huang Y.Y., de Arce V.J.B., Hamblin M.R. (2011). Animal models of external traumatic wound infections. Virulence.

[58] Ahmed S., Ikram S. (2016). Chitosan Based Scaffolds and Their Applications in Wound Healing. Achiev. Life Sci..

[59] Al-Roujayee A.S. (2017). Naringenin improves the healing process of thermally-induced skin damage in rats. J. Int. Med. Res..

[60] Abbas M.M., Al-Rawi N., Abbas M.A., Al-Khateeb I. (2019). Naringenin potentiated β-sitosterol healing effect on the scratch wound assay. Res. Pharm. Sci..

