# Supplementary material for: Development and Optimization of Naringenin-Loaded Chitosan-Coated Nanoemulsion for Topical Therapy in Wound Healing
Source: Pharmaceutics. 2020 Sep 20;12(9):893. doi: 10.3390/pharmaceutics12090893 (PMC7558164; doi:10.3390/pharmaceutics12090893)
Supplement: Supplementary file 1 [file pharmaceutics-12-00893-s001.pdf]

pharmaceutics

MDPI

Supplementary Materials: Development and Optimization of Naringenin-Loaded Chitosan-Coated Nanoemulsion for Topical Therapy in Wound Healing

Sabah H. Akrawi, Bapi Gorain, Anroop B. Nair, Hira Choudhury, Manisha Pandey, Jigar N. Shah and Katharigatta N. Venugopala

Table S1. Representation of mucoadhesive strength and force of adhesion of the formulated formulations on goatskin.

Formulation	Blank NE	Drug-loaded NE	0.5% CNNE	0.75 CNNE	1% CNNE
Mucoadhesive strength (g)	20.53 ± 1.22	21.5 ± 1.05	85.06 ± 1.26	93.06 ± 2.15	118.33 ± 2.08
Force of adhesion (N)	0.20 ± 0.017	0.21 ± 0.010	0.83 ± 0.012	0.91 ± 0.02	1.15 ± 0.02

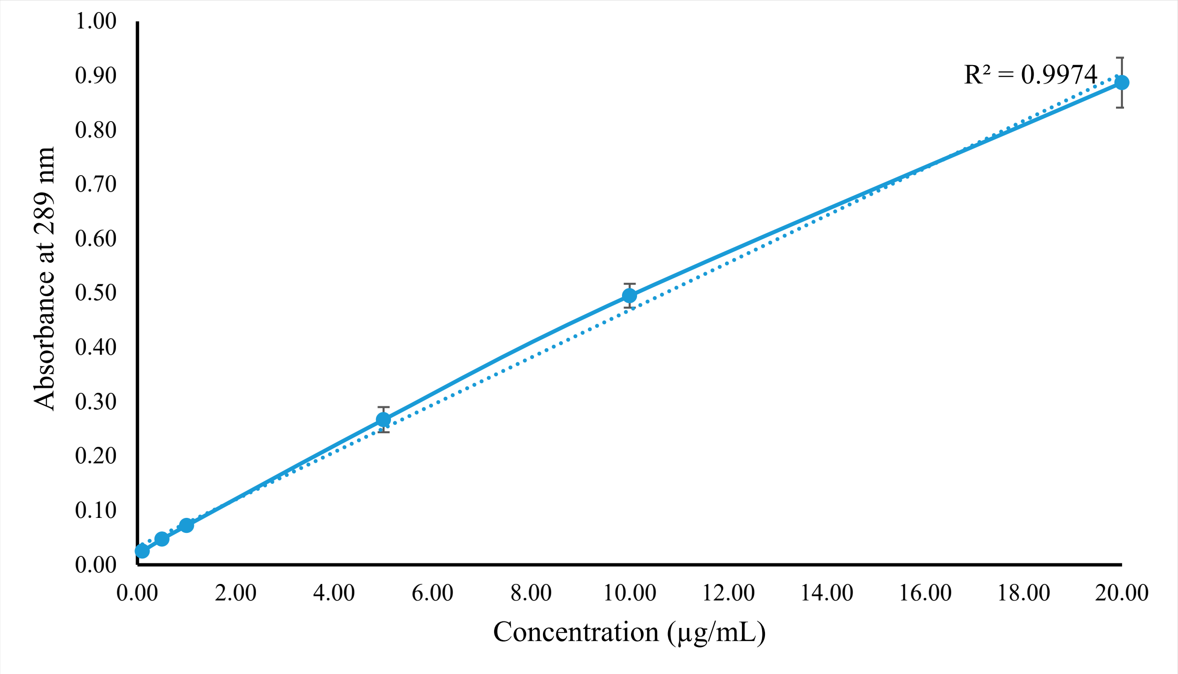

Figure S1. Calibration curve for determination of unknown concentration of naringenin using UV-visible spectrophotometer.

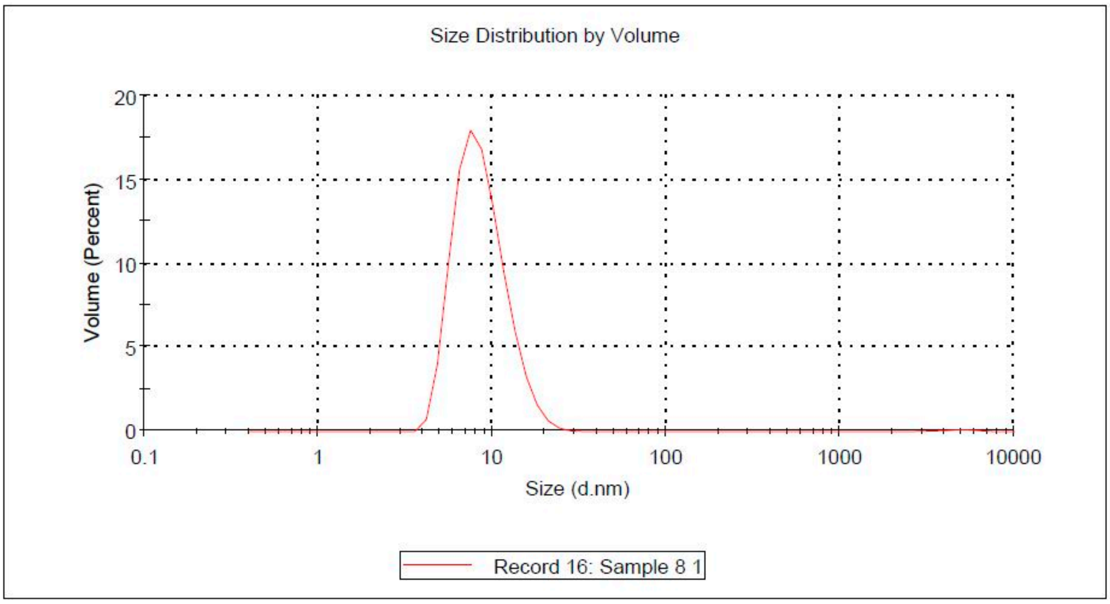

Figure S2. Representation of blank nanoemulsion particle size distribution.

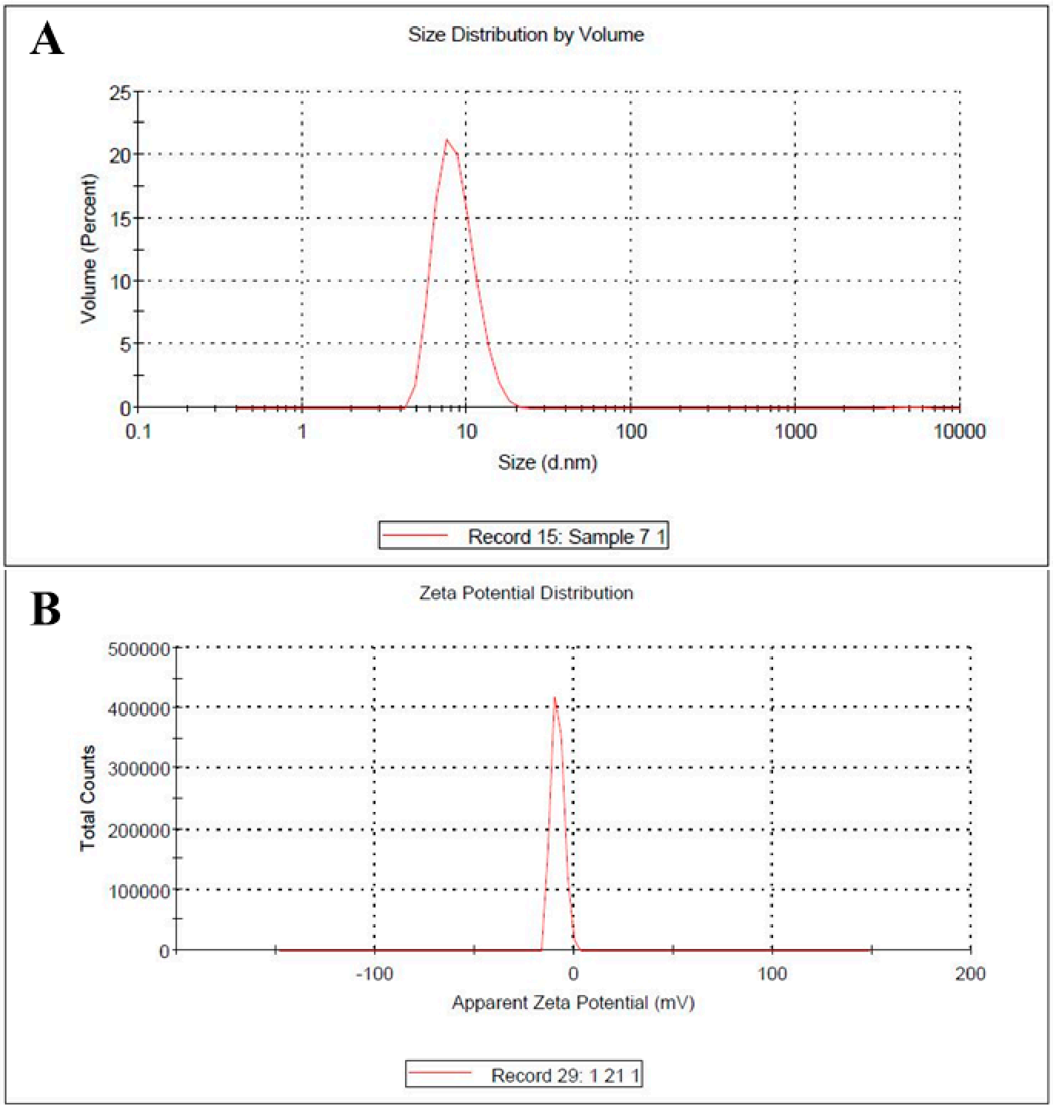

Figure S3. Representation of naringenin-loaded nanoemulsion particle size distribution (**A**) and zeta potential (**B**).
